# Transcriptional profile in rat muscle: down-regulation networks in acute strenuous exercise

**DOI:** 10.7717/peerj.10500

**Published:** 2021-04-02

**Authors:** Stela Mirla da Silva Felipe, Raquel Martins de Freitas, Emanuel Diego dos Santos Penha, Christina Pacheco, Danilo Lopes Martins, Juliana Osório Alves, Paula Matias Soares, Adriano César Carneiro Loureiro, Tanes Lima, Leonardo R. Silveira, Alex Soares Marreiros Ferraz, Jorge Estefano Santana de Souza, Jose Henrique Leal-Cardoso, Denise P. Carvalho, Vania Marilande Ceccatto

**Affiliations:** 1 Superior Institute of Biomedic Sciences, Universidade Estadual do Ceará, Fortaleza, Ceará, Brazil; 2 Digital Metropolis Institute, Universidade Federal do Rio Grande do Norte, Natal, Rio Grande do Norte, Brazil; 3 Institute of Biology, Universidade Estadual de Campinas, Campinas, São Paulo, Brazil; 4 Carlos Chagas Filho Biophysics Institute, Universidade Federal do Rio de Janeiro, Rio de Janeiro, Brazil

**Keywords:** Transcriptome, Soleus muscle, Acute strenuous exercise

## Abstract

**Background:**

Physical exercise is a health promotion factor regulating gene expression and causing changes in phenotype, varying according to exercise type and intensity. Acute strenuous exercise in sedentary individuals appears to induce different transcriptional networks in response to stress caused by exercise. The objective of this research was to investigate the transcriptional profile of strenuous experimental exercise.

**Methodology:**

RNA-Seq was performed with *Rattus norvegicus* soleus muscle, submitted to strenuous physical exercise on a treadmill with an initial velocity of 0.5 km/h and increments of 0.2 km/h at every 3 min until animal exhaustion. Twenty four hours post-physical exercise, RNA-seq protocols were performed with coverage of 30 million reads per sample, 100 pb read length, paired-end, with a list of counts totaling 12816 genes.

**Results:**

Eighty differentially expressed genes (61 down-regulated and 19 up-regulated) were obtained. Reactome and KEGG database searches revealed the most significant pathways, for down-regulated gene set, were: PI3K-Akt signaling pathway, RAF-MAP kinase, P2Y receptors and Signaling by Erbb2. Results suggest PI3K-AKT pathway inactivation by Hbegf, Fgf1 and Fgr3 receptor regulation, leading to inhibition of cell proliferation and increased apoptosis. Cell signaling transcription networks were found in transcriptome. Results suggest some metabolic pathways which indicate the conditioning situation of strenuous exercise induced genes encoding apoptotic and autophagy factors, indicating cellular stress.

**Conclusion:**

Down-regulated networks showed cell transduction and signaling pathways, with possible inhibition of cellular proliferation and cell degeneration. These findings reveal transitory and dynamic process in cell signaling transcription networks in skeletal muscle after acute strenuous exercise.

## Introduction

Physical exercise acts as stressing agent on skeletal muscle, promoting molecular adaptations which depend on the intensity and type of the exercise accomplished (Egan & Zierath, 2013). Strenuous exercise has been studied specially due to the intense muscular energetic demands. Intensive sessions of acute exercise may induce positive metabolic adaptations  (Zychowska et al., 2017) and improvement of cognitive capacity (Dinoff et al., 2017). However, in sedentary individuals or during intensive training applied without an adequate recovering period, such sessions may cause inflammation, oxidative cellular damage, protein degradation and even muscular lesion (Peake, Nosaka & Suzuki, 2005; Baumert et al., 2016; Mohamed, Lamya & Hamda, 2016).

There are many factors involved in the physiology of the skeletal muscle in response to the stress caused by acute exercise such as expression of genes, proteins, activation of signaling cascades and other processes (Egan et al., 2013; Pacheco et al., 2018). Regulation of gene expression and activation of specific metabolic pathways are fundamental for skeletal muscle remodeling (MacNeil et al., 2010; Keller et al., 2011) and its been studied in different time periods, which may be hours or days, in order to identify cellular adaptations occurring between the inflammatory process and muscular recovery  (Paulsen et al., 2012; Gjevestad, Holven & Ulven, 2015).

In this respect, post-genomic approaches have been used in the subject of exercise physiology (Sapp et al., 2017; McGee & Walder, 2017; Fu et al., 2019). Previous molecular evaluations characterized some ‘omic’ features from physical exercise, such as differentially expressed genes (DEGs). In a previous study we found 743 up regulated genes and 530 down regulated gene in different intensities and types of exercises, with some gene clusters connected by regulating nets (Pacheco et al., 2018). In a previous study with exhausting exercise model for rats in an adapted treadmill we evaluated the expression of pro-oxidizing and anti-oxidizing enzymes in the muscle. A period of 24 h, after the strenuous exercise session, seemed to produce a response of acute phase of gene transcription. The results showed attenuation of the oxidative stress and intensification in expression of gene PPARGC1*α*, as well as oxidative defense adaptations and differential gene expression according to the type of muscular fiber  (Alves et al., 2020). However, that and other studies of gene expression (Alves et al., 2020; Neubauer et al., 2014) did not evaluate the complete transcriptional response in the muscle after exhausting exercise, but instead only some specific genes.

In the present study we propose a far-reaching evaluation of the gene expression caused by the exhausting exercise in the skeletal muscle from rats, 24 h after the exercise, in order to investigate the global transcriptional response in the structural and functional remodeling of the skeletal muscle. Based on a transcriptomic assay (RNA-seq), we show some metabolic pathways affected and some candidate genes responsible for the muscular stress induced by the strenuous exercise.

## Methods

### Animal experiments

The study was approved by the Ethics for Animal Use Committee of the State University of Ceará—UECE (protocol number: 1592060/2014). Eight 2-months old male Wistar rats with average weight of 220–280 g, provided by the vivarium of the Superior Institute of Biomedical Sciences at UECE, were used. Animals were kept in cycles of light/dark (12 h/12 h), in a controlled temperature (22–25 °C) environment, with water and feed ad libitum. Animal refusal to physical activity was used as a criterion for pre-exclusions. None of the animals were excluded or euthanized before completing the experiments, only after the experiment animals were euthanized. All animal experiments were performed in accordance with the Guidelines from the National Council for Animal Experimentation Control from Brazil.

#### Strenuous acute exercise

The study comprised two experimental groups: Control group (C) and Trained group (T). Both C and T were familiarized in an adapted treadmill (INBRAMED-Porto Alegre, Brasil) for two weeks during the nocturnal period. Acclimation conditions were completed in 5 days/week: 1st week: 0.4 km/h, 5 min and 2nd week: 0.4 km/h, 10 min. Animals in the control treatment were submitted only to the acclimation period. This session was used for the animal familiarization to the experimental environmental conditions and to minimize the stress created by the exercise protocol (Kregel et al., 2006). After two weeks of adaptation, only the trained group (T) performed a single strenuous exercise session, also nocturnal period, consisting in 3 min running stages with constant load, with initial velocity at 0.3 km/h and 0.2 km/h increments between stages until the animals reached physical exhaustion, a period determined by the animal refusal to continue exercise and loss of limb coordination (Teixeira et al., 2012). An indicator of strenuous exercises is the occurrence of fatigue with ceric lactate accumulation. Our previous study demonstrated that a strenuous exercise session, with a protocol established by our study group, increases lactate levels in the animals (Alves et al., 2020).

#### Euthanasia and tissue collection

All animals, group C and T, were euthanized with Thiopental sodium (150 mg/kg) 24 h after the exhaustive exercise session. Samples of soleus muscle were desiccated and immediately immersed in RNA stabilizing solution (*RNAlater—RNA Stabilization Reagent/* QIAGEN, Hilden, Germany), following recommendations by the manufacturers. Stabilized samples were conditioned at −80 °C. Muscle samples were used for gene expression analyses by RNA-seq and RT-qPCR.

### Sequencing

#### RNA extraction and quantification

Extraction of total RNA was achieved with *TRIzol®* (Thermo Fisher Scientific/Massachusetts, EUA) and *RNeasy Plus Mini Kit*® (QIAGEN) following recommendations and specifications by manufactures. Integrity of the RNA in the samples was evaluated by capillary electrophoresis, using the *Agilent 2100 Bioanalyzer System* (*Agilent Tecnologies*/Santa Clara, CA, EUA). Quality of the extracted RNA was verified by the RNA integrity number (RIN). All samples demonstrated integral 18S and 28S bands. RNA concentrations obtained in the samples were of 466.6  ± 21.7 ng/µL and the 28S/18S ratio was of 1.4  ± 0.02. The RIN values obtained were 8.1  ± 0.14. Samples satisfied RNA quality and integrity requirements for sequencing.

#### cDNA libraries and sequencing

cDNA libraries were built with *Ilumina TruSeq Kit* (kits/truseq-rna-v2.html). Sequencing was performed using the *Illumina HiSEQ 2500 Platform* (*Illumina*, San Diego, CA, EUA). Sequencing coverage was of 30 million reads per sample, with 100bp paired-end sequenced reads.

### RT-qPCR

In order to validate gene expression results generated by RNA-seq analyses, six genes involved in muscle oxidative metabolism were submitted to relative expression analyses by RT-qPCR. Selected genes were: *Nox2, Nox4, Sod1, Sod2, Mstn* and *Cic.* Gene expression quantitative analysis was performed using the *Bio-Rad CFX96* system (*Bio-Rad*, CA, EUA). Assays were performed in duplicate using 2µL of previously synthetized cDNA, adding to the mix a reaction of 10µL of *Power SYBR Green* (*Life technologies*, CA, EUA), 6.8µL ultra-pure water (*Life Technologies*, CA, EUA) and 0.6 *μ*L (300 nM) of each primer (forward and reverse) (Supplemental Material Fig. S1_Validation of RNA-seq by RT-qPCR https://doi.org/10.6084/m9.figshare.9738203.v2).

### Bioinformatics analyses

#### Processing RNA-seq sequences obtained

Sequenced read quality was evaluated using FastQC v0.11.4 adopting standard parameters (Andrews, 2010). Removal of adaptors and filtration of low-quality sequences were performed with *Trim Galore* v0.4.1  (Martin & Wang, 2011) and *Cutadapt* v1.8.3 (Krueger, 2015). The sequencing data showed in the present study have been submitted to the National Center for Biotechnology Information’s Sequence Read Archive (SRA) and are accessible through BioProject PRJNA557195.

#### Mapping with reference genome

After low quality sequences were removed, reads sequences were mapped with reference genome (Rno 4.0) using TopHat v2.1.0 (Trapnell, Pachter & Salzberg, 2009) following -G ref_genes.gtf parameters. Genome version used was Rno 4.0 with transcriptome version dated 27/01/2011, both obtained in the UCSC Genome Browser (https://genome.ucsc.edu/). Sequence ordination was performed with Samtools v1.7  (Li et al., 2009) and the resulting files were used as inputs to estimate transcript relative abundance.

#### Transcripts identification and quantification

Reads sequences, previously selected with the required quality, were mapped with non-redundant reference transcriptome, to estimate the abundance of transcripts and isoforms using *TopHat* standard parameters. Transcript quantification for each identified gene was performed using HTSeq v0.11.0 (Anders, Pyl & Huber, 2015). Ambiguous and chimeric read sequences were not computed, beyond that, sequences were quantified with MAPQ (*Mapping quality score*) alignment above 10. These analyses generated a table with all the identified genes and its respective mean expression values for each sample, and this was the input for differential gene expression analyses.

#### Differential gene expression analyses

Gene expression analyses were completed with *EBseq* v 1.22.1  (Leng et al., 2013) package in R (Version 3.5). The transcript quantification data was evaluated following *Fold change* ≥ 1.4, *p* ≤ 0.05 and FDR ≤ 0.05 parameters.

#### Gene ontology and enrichment

The set of differentially expressed genes was analyzed for gene ontology enrichment and identification of the metabolic pathways affected, with Database for Annotation, Visualization and Integrated Discovery—DAVID platform, version 6.9 (Huang, Sherman & Lempicki, 2009). DEGs were analyzed in three Gene Ontology—GO (Mi et al., 2017) categories: molecular function (MF), cellular component (CC) and biological process (BP). Metabolic pathway analysis was performed using the Kyoto Encyclopedia of Genes and Genomes—KEGG (Kanehisa et al., 2012), Reactome (Fabregat et al., 2018) with DAVID *Functional Annotation Tool*, parameters: count genes ≥ 2, EASE (Modified Fisher Exact *p*-value for gene-enrichment analysis): 0.1, *p* ≤ 0.05. The gene set was separated into two subsets, up- and down-regulated, for gene ontology and enrichment analyses.

## Results

### Differential expressed genes (DEGs)

**Figure 1:**
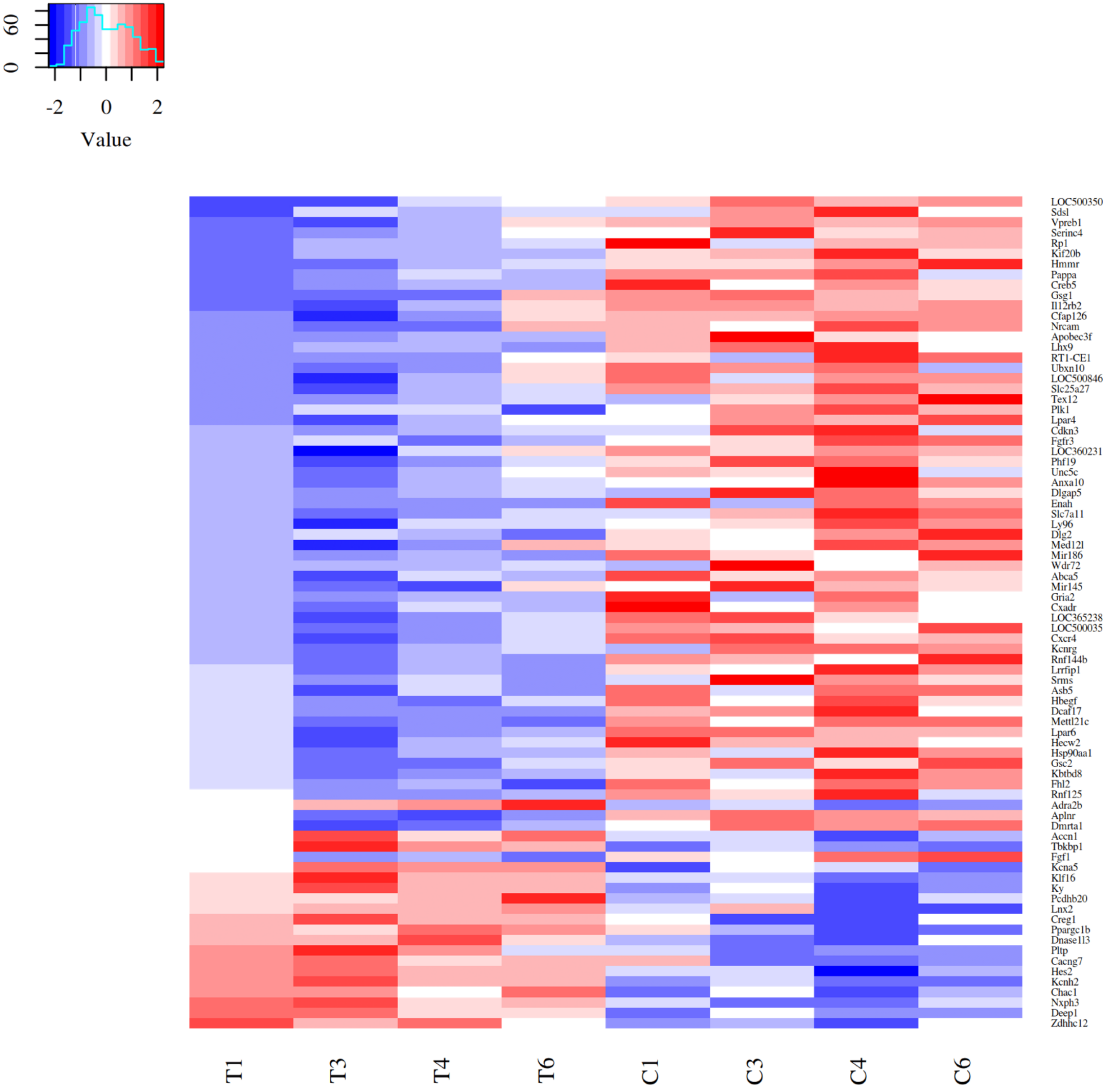
Heatmap of 80 DEGs expression patterns in acute strenuous exercise from rat soleus muscle. Comparison of gene expression profiles between control (C1–C6) and trained (T1–T6) groups. For each gene, the relative values of gene expression are represented by the blue and red tones, where blue indicates less expression (down regulated—61 genes) and red greater expression (up regulated—19 genes).

Differential expressed genes (DEGs) after acute strenuous exercise in soleus muscle of rats were obtained following the parameters: FC ≥ 1.4, FDR ≤ 0.05 and *p* ≤ 0.05. A heat map of 80 resulting DEGs with 19 up-regulated and 61 down-regulated genes was obtained (Fig. 1), with *log2fc* values from 0.14 to 2.59. The most significant differential expression values are showed in Top20 DEGs (Table 1). Up-regulated expressive DEGs were: *Chac1* (*log2fc* 1.37), *Asic2* (1.36), *Hes2* (1.11), *Loc500300* (1.04) and *Ky* (0.90), while down-regulated DEGs included *Lhx9* (*log2fc* −2.79), *Wdr72* (−2.40), *LOC500035* (−2.04), *Gsc2* (−1.84) and *Dlg2* (−1.68). Transcription factors observed in the Top 80 DEGs set were *Creg1* (*log2fc* 0.52), *Ppargc1b* (0.53), *Klf16* (0.61), *Hes2* (1.11), all up-regulated, and *Lhx9* (−2.79), *Mettl21c* (−1.45) and *Cxcr4* (−0.53), down-regulated factors (Supplemental Material Table S1_TOP 80DEGs https://doi.org/10.6084/m9.figshare.9738278.v2).

**Table 1:** Top 20 differentially expressed genes after strenuous acute exercise in soleus muscle in rats. The genes with the highest fold change values (≥1.4) expressed after strenuous acute exercise in soleus muscle in rats, presented by log2FC, 10 up regulated and 10 down regulated.

**Symbol**	**Ensembl**	**Gene**	***log2fc***
***Up-regulated*genes**			
Chac1	ENSRNOG00000014387	ChaC glutathione-specific gamma-glutamyl cyclo transferase 1	1.38
Asic2	ENSRNOG00000058308	Acid sensingion channel subunit 2	1.37
Hes2	ENSRNOG00000010490	Hes family b HLH transcription factor 2	1.11
Deep1	ENSRNOG00000023465	DEPP1, autophagy regulator	1.05
Ky	ENSRNOG00000008210	Kyphoscoliosis peptidase	0.90
Kcna5	ENSRNOG00000019719	Potassium voltage-gated channel subfamily A member 5	0.89
Cacng7	ENSRNOG00000056257	Calcium voltage-gated channel auxiliary subunit gamma 7	0.71
Adra2b	ENSRNOG00000013887	Adrenoceptor alpha 2B	0.64
Klf16	ENSRNOG00000033694	Kruppel-likefactor 16	0.61
Dnase1l3	ENSRNOG00000009291	Deoxyribonuclease 1-like 3	0.59
***Down-regulated genes***			
Lhx9	ENSRNOG00000010357	LIM homeobox 9	−2.79
Wdr72	ENSRNOG00000054889	WD repeat domain 72	−2.40
LOC500035	ENSRNOG00000031207	Hypothetical protein LOC500035	−2.04
Gsc2	ENSRNOG00000000282	Goose coid homeobox 2	−1.85
Dlg2	ENSRNOG00000022635	Discs large MAGUK scaffold protein 2	−1.68
LOC500350	ENSRNOG00000030158	LRRGT00139	−1.65
Cdkn3	ENSRNOG00000009785	Cyclin-dependent kinase inhibitor 3	−1.54
Sdsl	ENSRNOG00000001391	Serine dehydratase-like	−1.48
Mettl21c	ENSRNOG00000011591	Methyltransferaselike 21C	−1.45
Tex12	ENSRNOG00000049470	Testis expressed 12	−1.36

### Ontology and enrichment

**Figure 2:**
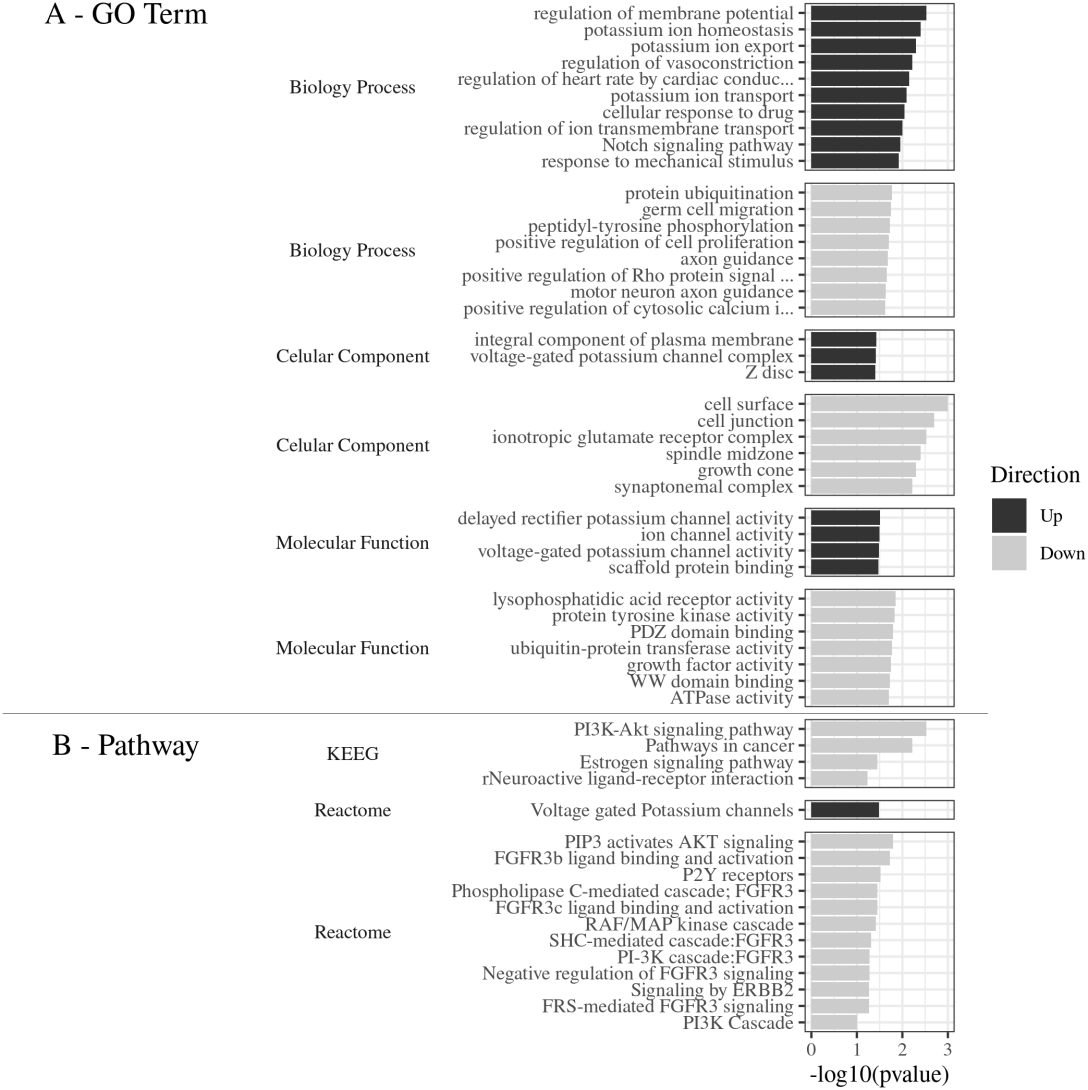
(A) GO Term. Functional classification of DEGs for the Gene Ontology categories. (B) Pathways. The most significant metabolic pathways identified by KEGG and Reactome. (A) GO Term The 80 DEGs, divided into up and down regulated, were evaluated according to the 3 GO categories: Biology process, Cellular component and Molecular function. (B) Pathways. The most significant metabolic pathways, for the 80 DEGs, were identified by KEGG and Reactome, through DAVID, with the parameters: count ≥2, EASE: 0.1, *p* ≤ 0.05, presented by −log 10 (*p* value).

Gene Ontology of the top 80 DEGs was categorized in three categories: biological process (BP), cellular component (CC), and molecular function (MF). The DEGs were classified into 38 categories of the *R. norvegicus* genome. About 21 GO terms were related to down-regulated genes and 17 to up-regulated genes (Fig. 2A). The up-regulated DEGs revealed the terms: cell surface (eight genes), cell junction (six), ionotropic glutamatergic receptor complex (two), and others, totaling six terms for 16 non-redundant genes. In the category of biological process, the most enriched terms were: protein ubiquitination (five), peptidyl-tyrosine phosphorylation (three), germ cell migration (two), positive regulation of cell proliferation (two), and positive regulation of cytosolic calcium ion concentration involved in phospholipase C-activating G-protein coupled signaling pathway (two), 8 terms were identified for 14 non-redundant genes. The molecular function was represented by the following terms: lysophosphatidic acid receptor activity (two genes), protein tyrosine kinase activity (three), PDZ domain binding (three), ubiquitin-protein transferase activity (four), WW domain binding (two), and ATPase activity (two), with a total of seven terms for 17 non-redundant genes. (Supplemental Material Table S2_Complete Gene Ontology for functional classification of DEGs: https://doi.org/10.6084/m9.figshare.9738290.v3).

#### Metabolic pathways identification

The main pathways enriched for the down-regulated gene pool, according to the Reactome Database were: PIP3 activated AKT signaling (three genes), FGFR3b ligand binding and activation (two), P2Y receptors (two), Phospholipase C-mediated cascade FGFR3 (two), FGFR3c ligand binding and activation (two), RAF/MAP kinase cascade (three), SHC-mediated cascade FGFR3 (two), Negative regulation of FGFR3 signaling (two), PI-3K cascade FGFR3 (two), Signaling by ERBB2 (two), FRS-mediated FGFR3 signaling (two), and PI3K Cascade (two), in a total of 12 pathways and six non-redundant genes.

The most significant pathways in the KEGG database for the down-regulated gene set (Fig. 2B), were: PI3K-Akt signaling pathway (number of genes: six), pathways in cancer (six), Neuroactive ligand–receptor interaction (four), and estrogen signaling pathway (three), in a total of 4 pathways and 10 non-redundant genes.

The most significant metabolic pathway for the down-regulated gene pool was the PI3K-Akt signaling pathway, inferred by the KEGG and also by the Reactome databases, with a total of seven non-redundant genes (Lpar4, Fgfr3, Fgf1, Lpar6, Hsp90aa1, Creb5, and Hbegf).

(Supplemental Material Table S3_Metabolic pathways obtained by Reactome and KEGG databases for DEGs https://doi.org/10.6084/m9.figshare.9738422.v1). This results shows that 23.33% of the enriched DEGs were related to this specific metabolic pathway, with the −log10 (*pvalue*) value of 2.5 (Fig. 2B).

#### Metabolic pathway interactome

**Figure 3:**
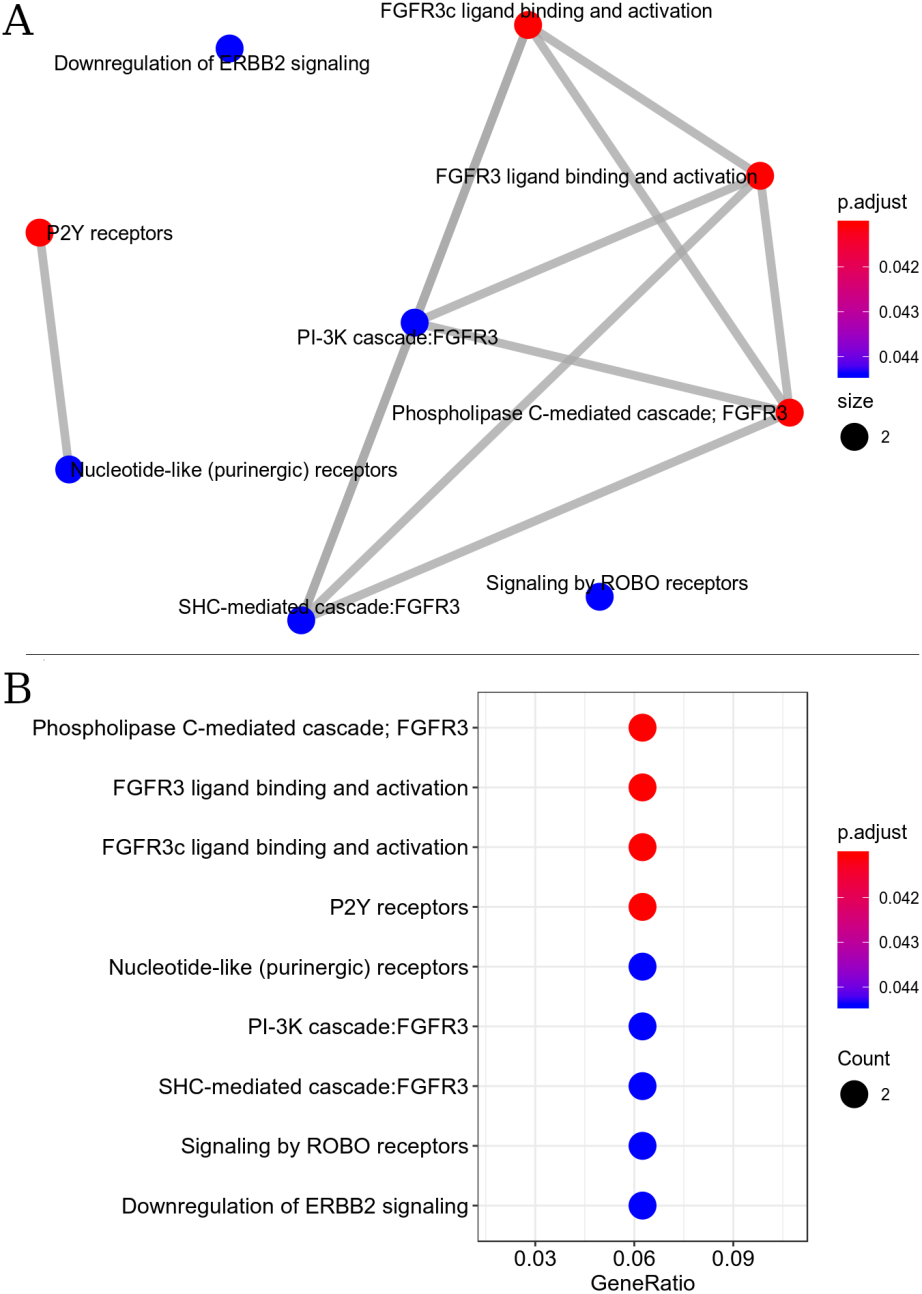
Interaction of metabolic pathways. The most significant metabolic pathways interconnected by the Reactome. (A) The most significant metabolic pathways, and linked by the Reactome, had genes in common indicating a possible inactivation of the PI3K/Akt pathway. The points represent the number of genes involved in the pathway, the color of the point indicates the *p* adjust value and the edges indicate the interaction between the pathways. (B) The most significant metabolic pathways with *p* adjust value.

The resulting metabolic interactome pathway interconnected by the Reactome database were: PIP3 activates AKT signaling, FGFR3b ligand binding and activation, FGFR3c ligand binding and activation, PI3K Cascade, PI-3K cascade, FGFR3, Phospholipase C-mediated cascade - FGFR3, SHC-mediated cascade, FGFR3, Negative regulation of FGFR3 signaling, FRS-mediated FGFR3 signaling (Fig. 3). This set of pathways triggers the activation/inactivation of the Phosphatidylinositol 3-kinase (PI3K)/Akt super-path.

Signal transduction pathway for the down-regulated set was found to be the most enriched super-path, represented by the following paths: PI3K-Akt signaling pathway, RAF/MAP kinase cascade, P2Y receptors and signaling by ERBB2. Enrichment analysis revealed that signal transduction pathways were the most affected by exercise.

## Discussion

The molecular mechanisms involved in the physiological adaptations of the skeletal muscle as a response to exercise are being considerably investigated in the last years. Many studies have evaluated the levels of gene expression in the skeletal muscle under different types of training and intensities of exercise (Egan & Zierath, 2013; Pacheco et al., 2018; Vissing & Schjerling, 2014). Levels of gene expression after acute exercise session regulate the respective content of proteins in the skeletal muscle (Egan et al., 2013; Perry et al., 2010) and that gene and protein expression varies with time, from hours to days. Acute exercise sessions activate signaling cascades and a transitory gene expression in the following hours after the exercise (Alves et al., 2020; Hoppeler, Klossner & Flück, 2007; Louis et al., 2007). Such transitory transcriptional response may activate mechanisms of cellular stress  (Perry et al., 2010; Yu et al., 2003; Pawlikowski et al., 2017) and cause events similar to inflammation in the muscle, few hours after exercise (Paulsen et al., 2012; Neubauer et al., 2014; Tidball & Villalta, 2010). However, the studies mentioned evaluated the gene expression in the muscle, after acute exercise, via genomic microarrays or RT-PCR, limiting the quantity and specificity of evaluated genes and does not show an ample transcriptional response. As far as we know, a complete transcriptome (RNA-seq) of the soleous muscle in rats 24 h after strenuous exercise session in treadmill has not been evaluated yet. The molecular aspects involved in the stress responses caused by an acute exercise session are not yet completely resolved. In the present work, we evaluate the complete transcriptional response of the skeletal muscle from rat 24 h after a session of strenuous exercise.

Cell response occurred mainly through signal transduction pathways: PI3K–AKT signaling pathway, Sinaling by Erbb2, RAF-MAP kinase and P2Y receptors. In addition, the Top20 DEGs comprehended genes associated with cellular stress induced by strenuous exercise.

### PI3K-AKT signaling pathway

A Gene Set Enrichment Analysis revealed the super pathway of PI3K–AKT signaling pathway as the most significant, with 5 interconnected pathways (Fig. 3) and 3 genes in common, Fgf1 (log2fc −0.81), Fgr3 (−0.55) and Hbegf (−0.97), both down regulated. These genes codify growth factors activating PI3k-Akt, Erbb2 and RAF-MAP kinase pathways. The skeletal muscle, as well as pancreas and adipose tissue, are involved in signaling of Fgfs genes. Different from the linking properties of the majority of Fgfs, the Fgf1 is able to link to isoforms ’b’ and ’c’ of proteins Fgfr1, Fgfr2, Fgfr3 and Fgfr4 (Goetz & Mohammadi, 2013; Yablonka-Reuveni et al., 2015). In our study, isoforms of genes Fgfr3b and Fgfr3c were detected, this seems to be a rare transcriptional profile of intracellular signaling in the muscle, once previous studies (Pawlikowski et al., 2017) showed the adult skeletal muscle tissue expresses only moderated levels of gene Fgfr1 in very low levels or even undetectable form the other subunits of Fgfrs. Inhibition of expression of genes Fgf1 and Fgr3 was followed by the low expression of genes PI3k (log2fc: −0.39) and Akt (−0.2). Consequently the inactivation of the PI3k-Akt way increases the levels of expression of apoptotic genes, such as Bcl2 (log2fc: 0.15) and Casp 9 (0.22), both also detected in our study. These findings suggest the inactivation of the PI3K-AKT way, regulated by the low expression of genes Fgf1 and the receptor Fgr3, which may contribute to inhibit cellular proliferation and increase apoptosis in response to the stress caused by the strenuous exercise session.

### Sinaling by Errb2

Another gene codifying a growth factor inhibited by the exhausting exercise was Hbegf (Heparin-biding EGF-like growth factor). Pathways related with this gene are PIP3 activates AKT signaling (R-RNO-1257604), RAF/MAP kinase cascade (R-RNO-5673001) and Signaling by Erbb2 (R-RNO-1227986). The skeletal muscle from rodents and adult humans, expresses the receptors ErbB2, ErbB3 and ErbB4 for member of the family EGF. Hbegf links to the receptor ErbB4 and induces its phosphorylation, activating the Phosphoinosithide 3-kinase, a component of the Akt signaling pathway. In addition, muscular contraction activates gene ErbB4, as well as the protein Akt (Jin et al., 2005; Citri, Kochupurakkal & Yarden, 2004).

These observations suggest Hbegf links to ErbBs in the cellular surface, therefore, activates the Phosphoinosithide 3-kinase-Akt signaling pathway. The study of Fukatsu et al. (2009), has demonstrated that moderated aerobic exercise increases expression levels of gene Hbegf in the soleous muscle of trained rats. However, our results show that, after an strenuous exercise session, the levels of Hbegf expression significantly reduced (log2fc −0.97), implying in more than one cell growth factor inhibited, which consequently generated a response of inactivation of PI3K-Akt and RAF-MAP kinase pathways. Nevertheless, is not yet totally clarified how the protein HBEGF contributes in muscular metabolism (Fukatsu et al., 2009).

### RAF-MAP kinase cascade

Physical exercise activates the pathway of RAF-MAP kinase in the muscle, culminating in cellular proliferation and muscular regeneration  (Rothfels, 2015). However, the reaction cascade, until phosphorylation of proteins ERKs and MEKs, increases in a dependent manner with the intensity of exercise (Widegren et al., 2000). Long-term resistance exercise may increase phosphorylation of MAPK3 and MAPK1 (ERK 1 and 2) of the skeletal muscle  (Lim & Kim, 2020) and acute exercise activates main MAPK subfamilies in human muscle  (Egan et al., 2013). In our model of strenuous exercise this pathway seems to be also inhibited by the low expression of genes Fgf1, Fgfr3 and Hbegf.

### Purinergic signaling

The strenuous exercise session also reduces the expression levels of genes Lpar4 and Lpar6. Purinergic receptors in the skeletal muscular system, expressed by satellite cells, may act in muscular excitability during intense exercises, as well as in the differentiation and repair of muscular cells (Burnstock, Arnett & Orriss, 2013). Positive regulation of Na+/K+/ATPase activity during muscular action seems to be associated with the stimulating effect of the adenosine 5′-diphosphate (ADP), acting through receptors P2Y (Walas & Juel, 2012). Signaling via P2Y1 receptors to increase the activity of the Na+/K+ pump, improves force and excitability of the depolarized skeletal muscle, which may be important to maintain excitability during intense exercises (Broch-Lips, Pedersen & Nielsen, 2010). Extensive research is under development to find modulator of P2Y receptors and to discover their physiological roles (Le Duc et al., 2017). The results showed in the present study suggest a negative regulation of the purineric signaling, showing that even 24 h after the exhausting exercise session, the metabolism activates pathways associated to cellular stress.

### Autophagy and apoptosis regulation genes

Gene Chac1 (Chac glutathione-specific gamma-glutamylcyclotransferase 1, RGD ID 1307153, log2fc 1.37), showed the highest value of fold change and is associated to the apoptosis process induced by stress. Genes Depp1 (autophagy regulator, RGD ID 1565700, log2fc 1.04) and Dnase1l3 (deoxyrribonuclease 1-like 3, RGD ID 620669, log2fc 0.59) are associated to autophagy regulation factors and linkage to damaged DNA, inducing apoptosis. Salcher et al. (2017) showed the protein coded by the gene Depp1 is located in the peroxisomes and mitochondria in the cells and interferes in the detoxification by reactive oxygen species (ROS), acting as a regulator of autophagy. Under conditions of cellular stress, high levels of cellular ROS contribute to induce autophagy. However, the detailed molecular mechanisms inducing autophagy via ROS are yet not fully comprehended (Filomeni, De Zio & Cecconi, 2015). In view of these findings and our results in the present study, gene Depp1 may be a candidate to response to cellular stress induced by strenuous exercises.

The gene Dnase1l3 encodes one endonuclese Ca2+/Mg2+ dependent, which catalyzes the cleavage of the single or double strand DNA and may have a role in DNA fragmentation in the apoptotic process. Some studies show that, immediately after exercise, the damage level to the DNA is not changed; this damage may appear hours or days after exercise (Niess et al., 1998).

**Figure 4:**
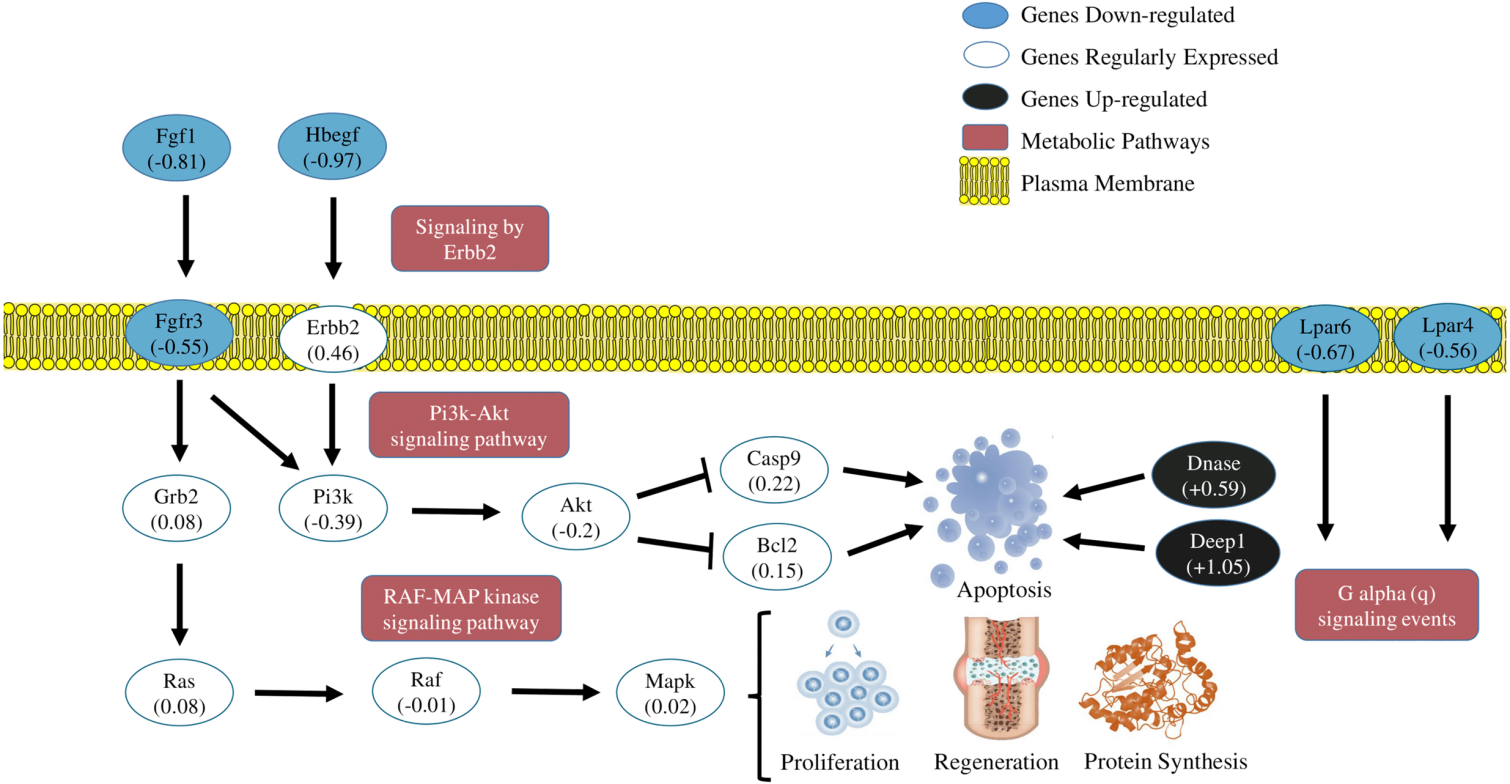
Transcriptional metabolic characterization model of PI3K-Akt/Erbb2/RAF-MAP kinase pathways. Signaling of the PI3K-Akt/Erbb2/RAF-MAP kinase pathways. The expression levels of the Fgf1, Hbegf, Fgr3, Lpar4, Lpar6 (blue-down regulated) and Dnase1 | 3, Depp 1 (back-up regulated) genes suggest an inhibition of cell proliferation and activation of apoptotic factors generated by stress caused by exercise. The other genes shown in the figure were detected in the sequencing, but did not show significant fold change values.

The transcriptome of the soleous muscle, in general, revealed a moment of cellular response induced by the acute exhausting exercise. Results suggest some metabolic pathways signaling cellular stress induced by exercise and the simultaneous activation of genes which code apoptotic and autophagy factors, in addition to the inactivation of metabolic pathways which inhibit apoptosis and regulate cellular proliferation, as can be observed in Fig. 4. Such findings reveal a transitory and dynamic response in the cellular signaling transduction networks of the skeletal muscle after acute strenuous exercise.

### Model of metabolic transcriptional characterization of the pathways PI3K-Akt/Erbb2/RAF-MAP/P2Y receptors

Summarizing the features of discussion, the transcriptional pattern of the main DEGs and metabolic pathways affected by the exhausting exercise, are represented in Fig. 4.

### Limitations of the study

Our study evaluated a specific period of the transcriptional response in the skeletal muscle of rats, 24 h after the strenuous exercise session in the treadmill. Other time periods were not considered; therefore this study does not reflect adequately all important alterations in the muscle transcriptome after acute exercise. Therefore, complementary analyses, with different time intervals need to be investigated in the future.

Despite the limited number of samples analyzed, the selected tool for gene expression analysis EBseq (Leng et al., 2013) is one of the five best performing tools, among 11 software analyzed (Schurch et al., 2016), preserving the false positive rate (FPR) near or lower that 5%, independently form the limit of fold change or the number of biological replicates, therefore being reliable for experiments with less than 12 samples.

To validate RNA-Seq results, some genes were selected for gene expression analysis via qRT-PCR. Results show RNA-Seq data are reliable and accurate. Still, using the same samples for RNA-Seq and qRT-PCR studies may have reduced the robustness of the results. Consequently, a new set of samples is required to replicate the genic expression data in future studies.

## Conclusions

Our findings aimed to elucidate the transcriptome of strenuous acute exercise in rat soleus muscle, implicating in a variety of dynamic cell processes. Networks obtained in gene set enrichment analyses predict strong links to cell transduction and signaling pathways, with negative regulation of cellular proliferation and cell degeneration. Apoptosis, DNA degradation and autophagy were the most notable predicted effects after strenuous acute exercise. These findings suggest a new point of view about strenuous acute exercise implications, allowing a physiological comprehension from molecular results.

##  Supplemental Information

10.7717/peerj.10500/supp-1 Supplemental Information 1 Correlation RNA-seq and RT-PCR

## Additional Information and Declarations

### Data Availability

The following information was supplied regarding data availability: The sequencing data is available at SRA: PRJNA557195.
